# Feature-Driven Semantic Communication for Efficient Image Transmission

**DOI:** 10.3390/e27040369

**Published:** 2025-03-31

**Authors:** Ji Zhang, Ying Zhang, Baofeng Ji, Anmin Chen, Aoxue Liu, Hengzhou Xu

**Affiliations:** 1 School of Mathematics and Statistics, Henan University of Science and Technology, Luoyang 471000, China; 230320080769@stu.haust.edu.cn (Y.Z.); baofengjihkd@163.com (B.J.); 220320080764@stu.haust.edu.cn (A.C.); 17739127399@163.com (A.L.); 2 Intelligent System Science and Technology Innovation Center, Longmen Laboratory, Luoyang 471000, China; 3 Engineering Research Center of Intelligent Swarm Systems, Ministry of Education, Zhengzhou University, Zhengzhou 450001, China; 4 School of Computer, Henan University of Engineering, Zhengzhou 450001, China; xhz05228@126.com

**Keywords:** semantic communication, non-uniform quantization, image feature extraction

## Abstract

Semantic communication is an emerging approach that enhances transmission efficiency by conveying the semantic content of information more effectively. It has garnered significant attention in recent years. However, existing semantic communication systems for image transmission typically adopt direct transmission of features or uniformly compress features before transmission. They have not yet considered the differential impact of features on image recovery at the receiver end and the issue of bandwidth limitations during actual transmission. This paper shows that non-uniform processing of features leads to better image recovery under bandwidth constraints compared to uniform processing. Based on this, we propose a semantic communication system for image transmission, which introduces non-uniform quantization techniques. In the feature transmission stage, the system performs varying levels of quantization based on the differences in feature performance at the receiver, thereby reducing the bandwidth requirement. Inspired by quantitative quantization techniques, we design a non-uniform quantization algorithm capable of dynamic bit allocation. This algorithm, under bandwidth constraints, dynamically adjusts the quantization precision of features based on their contribution to the completion of tasks at the receiver end, ensuring the quality and accuracy of the transmitted data even under limited bandwidth conditions. Experimental results show that the proposed system reduces bandwidth usage while ensuring image reconstruction quality.

## 1. Introduction

The global deployment and application of 5G technology have brought existing wireless communication systems closer to the Shannon limit in terms of performance [1]. This makes it difficult to further increase the channel capacity, and they cannot meet the massive and high-frequency data transmission demands under limited bandwidth conditions [2]. The development of artificial intelligence technology has provided new pathways for overcoming the limitations of traditional communication, driving the development of a communication paradigm aimed at transmitting semantic information–semantic communication. Compared to traditional communication, semantic communication emphasizes the transmission of information’s semantic content, thereby improving communication efficiency and task execution accuracy [3].

Current research mainly focuses on semantic communication for data types such as text, speech, and images. For example, Wang et al. [4] designed a semantic communication system for speech transcription based on the masked autoencoder; Xie et al. [5] combined visual and semantic data to design a semantic communication system for visual question answering. Hu et al. [6,7] used variational autoencoders to design a semantic communication system for image classification; Pan et al. [8] utilized semantic features from vehicle perception images to design an image segmentation semantic communication system for vehicle driving decision-making. However, these studies mainly focus on using the transmitted data to complete tasks at the receiver end, and they do not adequately address the issues related to data recovery and reconstruction, especially considering the high dimensionality and noise sensitivity of image data.

As a result, some researchers have focused on semantic communication systems for image transmission. E. Bourtsoulatze et al. [9] proposed an image transmission system based on convolutional neural networks (CNNs), which achieved better image recovery quality than traditional image transmission methods. K. Tonchev et al. [10] designed a semantic communication system based on graph convolutional networks (GCNs) to achieve the transmission and recovery of 3D video data. In addition to CNN-based communication systems, K. Yang et al. [11] used the self-attention mechanism and global modeling capability of transformers to design an image transmission system based on the Swin Transformer architecture, which showed superior image recovery performance compared to traditional communication systems and some semantic communication systems based on CNN architectures.

Although the image reconstruction results from the aforementioned semantic communication systems are satisfactory, they all aim to improve the quality of information recovery at the receiver end, with little consideration of the differential contribution of semantic information to the receiver.

This limitation has led to further consideration of how to optimize the transmission of semantic information, particularly how to apply differentiated encoding based on the contribution of different semantic information to the receiver. To address this, Lin et al. [12] proposed a semantic importance-based encoding algorithm that aggregates image channel features to obtain the importance weights of feature maps for task results, prioritizing the transmission of high-importance semantic feature maps to reduce redundant information in the transmission process. Considering the limited bandwidth issue in practical transmission, Huang et al. [13] divided the image into a base layer and an enhancement layer, prioritizing the transmission of key information at low bit rates to reduce transmission resource consumption. These approaches bear some resemblance to adaptive quantization methods in prior work. For instance, Wu et al. [14] proposed an adaptive quantization scheme based on semantic segmentation, dividing images into regions of interest (ROIs) and regions of non-interest (RONIs) with distinct encoding to optimize transmission efficiency. Gupta et al. [15] developed a method that dynamically adjusts quantization levels for neural network weights based on their statistical distribution, targeting memory reduction. Han et al. [16] introduced a technique combining pruning and quantization, adaptively assigning fixed bit-widths to weights based on magnitude for computational efficiency. Lei et al. [17] designed an ROI-based adaptive quantization for image compression, adjusting bit allocation to prioritize predefined regions critical for tasks like classification. However, these methods have notable limitations, that is, Wu et al. and Lei et al. relied on predefined ROI distinctions, restricting global optimization and adaptability to dynamic conditions; Gupta et al. and Han et al. applied uniform quantization across layers or parameters, neglecting feature-specific importance and bandwidth constraints during transmission.

In contrast, this paper proposes a feature-driven semantic communication framework that advances beyond these methods with non-uniform quantization based on feature contribution factors. Unlike Wu et al. [14] and Lei et al.’s [17] region-specific approaches or Gupta et al. [15] and Han et al.’s [16] uniform quantization, our method introduces a feature contribution factor to dynamically allocate bits across all image features according to their reconstruction importance. It optimizes global image recovery rather than predefined regions and adapts in real-time to bandwidth constraints, ensuring both efficiency and high-quality reconstruction under limited resources. The main contributions are as follows:

- We designed a feature-driven semantic communication framework that uses non-uniform quantization methods based on the contribution factor of image features, optimizing bandwidth usage while ensuring image quality.
- This paper designs a non-uniform quantization algorithm for transmitted features, which is based on fixed-point quantization technology. The algorithm dynamically adjusts the quantization precision by combining the contribution factors of features and the transmission bandwidth requirements, maximizing data transmission efficiency and image quality during the transmission process.
- The performance of the proposed system was validated under additive white Gaussian noise (AWGN), Rayleigh fading channels, and a composite channel model. This composite model combines Rician fading, multipath effects, and time-varying noise. The simulation results demonstrate the improvements in image transmission efficiency and quality of the proposed system compared to traditional uniform quantization. These improvements are particularly evident under limited bandwidth conditions across all tested channel environments.

The remainder of this paper is organized as follows: Section 2 describes the quantization techniques and the development of semantic communication. Section 3 presents the problem formulation, along with the system model and the design of the non-uniform quantization algorithm. Section 4 analyzes the existing simulation results. The conclusion is provided in Section 5, and Section 6 outlines potential directions for future work.

## 2. Related Works

### 2.1. Quantitative Methods

Quantization techniques were initially used for the digital processing of analog signals [18]. In particular, during the analog-to-digital conversion (ADC) process, continuous analog signals are discretized into a finite number of digital values, achieving a digital representation of the signal [19]. Quantization methods are divided into uniform and non-uniform quantization based on the difference in quantization intervals. Uniform quantization divides the data range into multiple equal-sized intervals for quantization, while non-uniform quantization performs quantization with unequal intervals based on the statistical properties of the data to improve quantization efficiency. In the field of image processing, quantization methods are widely applied in image compression, especially in early image compression standards such as JPEG [20], which uses uniform quantization techniques to quantize the coefficients after discrete cosine transform (DCT), adjusting the balance between compression ratio and image quality.

With the rise of deep learning and the increasing complexity of neural network models, quantization techniques have been redefined as an important method for neural network compression. Lin et al. [21] demonstrated the potential of quantization as a regularization technique by applying different bit quantization methods to multiple neural networks. Jacob et al. [22] proposed a pure integer quantization method, where both the weight and activation values were uniformly quantized to the INT8 type, further optimizing network performance. Notably, Liu et al. [23] introduced the binary neural network (BNN) quantization method, where all network weight and activation values are quantized to +1 and −1, significantly simplifying the model structure and reducing storage requirements. Inspired by this method, subsequent research has proposed various improvements and optimization methods [24].

In recent years, to improve the flexibility and applicability of quantization methods, mixed-precision quantization based on parameter differences has garnered significant attention [25]. This approach, which we refer to as a method of non-uniform quantization, allocates different bit precisions to parameters in different layers.

Chen et al. [26] designed a deep neural network model compression algorithm based on a three-value weight network, where full-precision weights were quantized into four states, enhancing the flexibility of weight quantization. Zhen et al. [27] introduced Hessian-aware quantization (HAWQ), which analyzes the curvature information of network layer outputs to establish a quantization sensitivity evaluation model, allocating the optimal bit width for each layer. Similarly, Zhang et al. [28] proposed a mixed-precision quantization method for depth-separable convolutional networks, assigning different bit widths to each layer based on the weight distribution and importance of each layer to reduce model size.

Uhlich et al. [29] proposed a method where the dynamic range and step size of the quantizer were parameterized as differentiable variables. By dynamically updating these parameters during training, they achieved the optimal bit width for mixed-precision quantization. In the ImageNet task, this method achieved less than 0.5% accuracy loss while compressing the ResNet-18 model by 4.24 times. Wu et al. [30] proposed the differentiable neural architecture search (DNAS), where a supernetwork with different bit-width candidate operations is constructed. By relaxing discrete bit-width choices to continuous probability distributions using Gumbel-Softmax, they assigned lower precision to redundant layers and retained high precision for sensitive layers, achieving model compression.

Wang et al. [31] proposed the APQ framework, which uses an evolutionary algorithm to search for mixed-precision strategies on a pre-trained “Once-For-All” network. By estimating latency and energy consumption for each layer at different bit-widths, the framework quickly computes model accuracy and resource usage, enabling pruning and mixed-precision quantization. Van et al. [32] introduced a Bayesian quantization framework that decomposes weights into multiple sets of power-of-two bit-width residuals. Using a variational inference approach, they jointly optimized bit-width and pruning gates, achieving mixed-precision quantization.

Additionally, some works have utilized reinforcement learning methods to dynamically select the appropriate quantization bit-width for each layer of neural networks. This helps maintain model inference accuracy while reducing storage and computational resource consumption. For example, Wang et al. [33] used the deep deterministic policy gradient (DDPG) to optimize per-layer bit-widths, incorporating hardware feedback (e.g., delay, energy consumption) to achieve multi-objective constraint optimization. In MobileNet-V2, this method achieved 8.03x model compression with only a 1.14% accuracy loss, considering FPGA resource constraints. Elthakeb [34] et al. proposed an LSTM-based reinforcement learning framework with an asymmetric reward function to balance accuracy and compression, prioritizing high precision for key layers and avoiding model collapse due to low precision.

Liu et al. [35] introduced a hierarchical reinforcement learning architecture (HLC and LLC controllers), jointly optimizing network architecture and per-core mixed precision. Using hardware model predictions, they reduced real deployment costs. This method, when applied to ResNet-50, reduced latency by 39% and energy consumption by 33%, compared to traditional methods. In summary, the development of non-uniform quantization techniques has greatly improved the efficiency of neural network models, enabling them to maintain high accuracy while reducing storage and computational demands.

### 2.2. Semantic Communication

As early as 1953, Weaver introduced the concept of semantics [36]. It focuses on transmitting semantic information rather than raw data [37,38]. Early communication theory mainly focused on the reliability of channel transmission. It paid limited attention to the semantics of the transmitted information [39]. However, with evolving communication needs [40] and advances in deep learning technology [41], semantic communication systems have gained increasing attention. Traditional communication systems focus on the accurate transmission of data. They do not consider the semantic relevance of the transmitted information. In contrast, using semantic encoding modules to extract and compress semantic features can improve communication efficiency and optimize the process [42,43,44,45]. Yet, many existing semantic communication systems face challenges in information transmission. These include high model complexity [46], large computational resource demands [47], and limited transmission efficiency.

To address these issues, some researchers have explored importance-based encoding. This approach aims to prioritize and encode information features based on their importance. It seeks to enhance transmission efficiency and reduce redundant data. These methods typically rank features and prioritize the transmission of higher-ranked ones. Zhou et al. [48] used feature importance and robustness as priority metrics. They prioritized the transmission of top-ranked features to ensure key image features are sent even under poor channel conditions. Gao et al. [49] defined semantic value to measure the importance of semantic information. They selected top-ranked semantic triples for transmission. Ma et al. [50] applied generative artificial intelligence (GenAI) to divide visual data into semantic segments. They ranked these segments by task relevance and transmitted only those with higher priority. Although these methods work in some cases, they generally face issues. These include high computational overhead, poor real-time performance, and complexity in dynamically assessing feature importance. In bandwidth-limited or resource-constrained environments, efficiently transmitting key features without adding computational burden remains a critical challenge. To address this, we propose a semantic communication framework based on feature contribution factors and non-uniform quantization. Compared to existing methods, our framework avoids complex feature ranking and predictive computation. It adjusts quantization precision according to each feature’s contribution to image recovery quality. Our method can adapt to the varying impacts of features on image reconstruction.

## 3. Feature-Driven Semantic Communication System

In this section, we discuss our proposed method in detail. First, we provide a theoretical analysis to demonstrate that, under resource-constrained conditions, applying non-uniform quantization to features, as opposed to uniform quantization, leads to better image reconstruction quality. Next, we introduce the semantic communication system model that we have designed. Finally, we elaborate on the non-uniform quantization method for features that we developed and explore its application in image reconstruction.

### 3.1. Problem Formulation

In communication systems, to reduce transmission bandwidth, quantization is applied to the extracted features before transmission. This process discretizes continuous-valued features, reducing data size while maintaining essential information. In semantic communication systems for image transmission, it is commonly assumed that all features contribute equally to image reconstruction at the receiver. Under this assumption, uniform quantization is applied to all features, treating them as equally important. However, in practice, different features have varying levels of significance. Some features play a more critical role in enhancing image quality, while others contribute less. Ignoring the non-uniform impact of features and applying uniform quantization can lead to the following issues:

1. Information loss: Important features may suffer excessive quantization errors, resulting in significant degradation of image quality.
2. Inefficient resource allocation: Less important features may receive unnecessary bit allocation, leading to inefficient transmission.

To address these issues, this paper proposes a feature-aware non-uniform quantization method that adapts quantization parameters based on the varying contributions of features. We introduce the contribution factor, $c_{i}$, to measure the importance of each feature in the image reconstruction. It is defined as follows:

(1) $$c_{i}=\frac{r(x_{i})}{\sum_{i=1}^{n}r\left(x_{i}\right)},$$

where $c_{i}$ represents the contribution factor of feature $x_{i}$, *n* denotes the total number of features, and $r(x_{i})$ quantifies the impact of feature $x_{i}$ on image reconstruction quality. Our objective is to design an adaptive quantization algorithm that optimizes transmission efficiency while maintaining high image recovery quality. In the next section, we analyze the advantages of non-uniform quantization in reducing quantization error.

### 3.2. Analysis of the Advantages of Non-Uniform Quantization in Image Recovery

Through analysis, we demonstrate the advantages of non-uniform quantization over uniform quantization in image recovery quality under a fixed bit budget.

#### 3.2.1. Assumptions

To facilitate the theoretical analysis, we make the following assumptions:

Assumption 1 (feature distribution): The extracted features $X=\{x_{1},x_{2},\ldots ,x_{N}\}$ are assumed to have a smooth probability distribution within their dynamic ranges. While deep neural networks may produce feature distributions that are approximately Gaussian or slightly non-uniform, we assume that within each feature’s dynamic range, the values can be approximated as locally uniform. This assumption can simplify the quantization error model and allow for an analytical derivation of the mean squared error (MSE).

Assumption 2 (quantization as the sole source of distortion): We assume that quantization is the sole source of distortion in the system, excluding factors such as channel noise or encoding errors.

Assumption 3 (bit budget constraint): We assume that the total available bits for quantization, denoted by *R*, is the sum of the bit allocations $b_{i}$ for each feature $x_{i}$, as follows:

$$R=\sum_{i=1}^{N}b_{i},\;\mathrm{where}\;b_{i}\in Z.$$

Additionally, the total bit budget is constrained by the maximum channel capacity $R_{\max}$, such that we have the following:

$$R\leq R_{\max}.$$

Assumption 4 (mean squared error as the quality metric): We use mean squared error (MSE) as the metric for evaluating image reconstruction quality, defined as follows:

$$MSE=\frac{1}{N}\sum_{i=1}^{N}{\left(x_{i}-{\hat{x}}_{i}\right)}^{2},$$

where $x_{i}$ represents the original image and ${\hat{x}}_{i}$ denotes the reconstructed image.

Assumption 5 (quantization error model): We assume that the quantization error for each feature $x_{i}$ follows a uniform distribution within its respective quantization interval:

$$\varepsilon_{i}=x_{i}-{\hat{x}}_{i}∼\mathrm{Uniform}\left(-\frac{\Delta_{i}}{2},\frac{\Delta_{i}}{2}\right),$$

where $\Delta_{i}$ is the quantization step size.

With these assumptions, we have the following theorem.

#### 3.2.2. Theorem and Proof

**Theorem 1.**  *Under Assumptions 1–5, the optimal bit allocation for minimizing the Mean Squared Error (MSE) in image recovery follows a non-uniform quantization scheme. The number of bits $b_{i}$ assigned to each feature $x_{i}$ is proportional to the logarithm of its contribution factor $c_{i}$, given by the following:*

(2) $$b_{i}^{*}=\frac{1}{2}\log_{2}\left(\frac{2c_{i}\cdot C\cdot \ln2}{\lambda }\right).$$

*Furthermore, non-uniform quantization results in a lower overall MSE compared to uniform quantization:*

(3) $$MSE_{\mathrm{non-uniform}}\leq MSE_{\mathrm{uniform}}.$$

**Proof.**  **Step 1: Formulating the MSE Expression** According to Assumption 5, the quantization error $\varepsilon_{i}$ follows a uniform distribution in its quantization interval, leading to a quantization noise variance:

(4) $$E\left[\varepsilon_{i}^{2}\right]=\frac{\Delta_{i}^{2}}{12}.$$

By Assumption 4, the total MSE is given by the following:

(5) $$MSE=\sum_{i=1}^{N}c_{i}\cdot \frac{\Delta_{i}^{2}}{12}.$$

Substituting the quantization step size, we have the following:

(6) $$\Delta_{i}=\frac{x_{i,\max}-x_{i,\min}}{2^{b_{i}}-1},$$

we obtain the following:

(7) $$MSE=\sum_{i=1}^{N}c_{i}\cdot \frac{{\left(x_{i,\max}-x_{i,\min}\right)}^{2}}{12(2^{2b_{i}}-1)}.$$

**Step 2: optimization problem formulation.** The objective is to minimize the total MSE subject to the bit budget constraint given in Assumption 3:

(8) $$\sum_{i=1}^{N}b_{i}=R.$$

Using the Lagrange multiplier method, we define the Lagrangian function as follows:

(9) $$L=\sum_{i=1}^{N}c_{i}\cdot \frac{{\left(x_{i,\max}-x_{i,\min}\right)}^{2}}{12(2^{2b_{i}}-1)}+\lambda \left(\sum_{i=1}^{N}b_{i}-R\right).$$

Following the standard derivation using the Lagrange multiplier method, the optimal bit allocation is obtained as follows:

(10) $$b_{i}^{*}=\frac{1}{2}\log_{2}\left(\frac{2c_{i}\cdot C\cdot \ln2}{\lambda }\right).$$

**Step 3: Performance Comparison** Applying the arithmetic-geometric mean (AM-GM) inequality, we have the following:

(11) $$\frac{1}{N}\sum_{i=1}^{N}a_{i}\geq {\left(\prod_{i=1}^{N}a_{i}\right)}^{\frac{1}{N}},$$

we establish the following:

(12) $$MSE_{\mathrm{non-uniform}}\leq MSE_{\mathrm{uniform}}.$$

This completes the proof.  □

The above theoretical analysis establishes that the proposed non-uniform quantization strategy effectively reduces MSE compared to uniform quantization. Equation (16) indicates that the number of bits assigned to each feature depends on its relative importance, leading to a more efficient representation of the image data. This result ensures reduced storage and transmission bandwidth requirements while maintaining image recovery quality.

### 3.3. Overall Architecture

**Figure 1:**
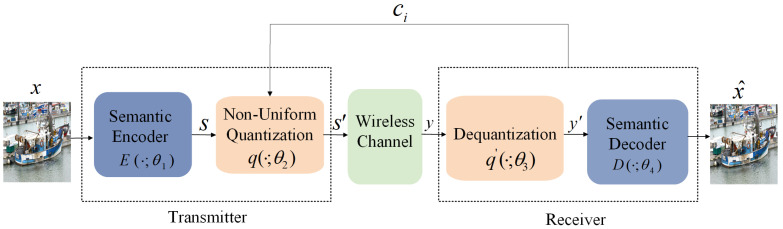
The overall architecture diagram of the proposed non-uniform quantization semantic communication system.

Based on the above theory, this paper designs a feature-contribution-factor-based non-uniform quantization semantic communication system, which reduces data storage and transmission bandwidth requirements while ensuring image recovery quality. The semantic communication system considered in this paper consists of two main parts, namely, the transmitter and the receiver. The system flow is shown in Figure 1.

The transmitter extracts features from the input image, *x*, through the encoder, *E*, and obtains the encoded feature vector, *s*; the transmission process is represented as follows:

(13) $$s=E(\cdot ;\theta_{1}),$$

where $\theta_{1}$ represents the parameter set of the encoder. Based on the contribution factors of the features, the representation of the feature *s* is non-uniformly quantized to obtain the compressed feature $s^{\prime }=q\left(\cdot ;\theta_{2}\right)$, where $\theta_{2}$ is the parameter set used for non-uniform quantization. Before transmitting the compressed feature through the wireless channel, power normalization is performed to ensure that the signal satisfies the specified average power constraint. In this paper, we consider a general fading channel model, where the transmission process is represented as follows:

(14) $$y=h⊙s^{\prime }+n,$$

where *h* denotes the channel state information (CSI) vector, representing the channel characteristics, ⊙ denotes element-wise multiplication, and *n* is the noise vector, with each component independently sampled from a Gaussian distribution. The noise distribution is characterized by noise power $\sigma_{n}^{2}$, and $I_{k}$ is the identity matrix. The receiver performs the corresponding dequantization on the received feature vector *y* to obtain $y^{\prime }=q^{\prime }\left(\cdot ;\theta_{3}\right)$. The decoder *D* is then used to reconstruct the dequantized feature into the image $\hat{x}$, which can be expressed as follows:

(15) $$\hat{x}=D\left(\cdot ;\theta_{4}\right),$$

where $\theta_{3}$ represents the parameter set for dequantization, and $\theta_{4}$ represents the parameter set of the decoder.

### 3.4. Non-Uniform Quantization Algorithm Based on Feature Contribution Factors

**Figure 2:**
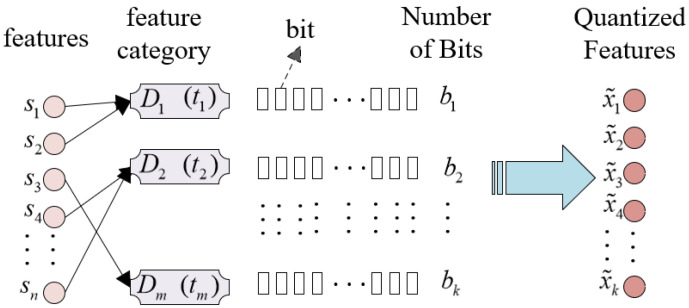
The overall architecture of the proposed non-uniform quantization.

This section designs a non-uniform quantization algorithm based on feature contribution factors. The algorithm can assign differential quantization precision to image features based on their contribution, maintaining the integrity of key information while reducing the data size, lowering transmission cost, improving transmission efficiency, and facilitating compatibility with digital communication systems. This foundation optimizes bandwidth utilization for efficient data transmission in practical communication environments. The core idea of the algorithm is to first classify features into different categories based on their contribution factors. Then, the total bit count is allocated to each category of features, followed by the allocation of bits to the symbol, integer, and fractional parts, and calculating the quantization step sizes for each part. As depicted in Figure 2, the *n* features after semantic encoding are divided into *m* feature categories based on their contribution factors, denoted as $D_{1},D_{2},\ldots ,D_{m}$. Each category contains $t_{1},t_{2},\ldots ,t_{m}$ features, and their sum equals *n*. For each feature category $D_{j}$, the total number of bits allocated for the feature quantization representation is denoted as $b_{k}$, where *k* represents the feature category, and quantization is performed according to a specific bit allocation formula.

First, based on the feature contribution factors, the sender classifies the features into priority categories before transmission. The larger the contribution factor, the higher the priority of the corresponding feature. These features are divided into *m* categories according to their priority. For each feature category $D_{j}$, the total number of bits allocated for feature quantization representation is denoted as $b_{k}$, where *k* represents the feature category. The bit allocation formula is defined as follows:

(16) $$b_{k}=\left\lfloor \frac{\sum_{i=1}^{k}c_{i}}{\sum_{k=1}^{M}\sum_{i=1}^{k}c_{i}}\cdot B\right\rfloor$$

where *B* is the total bit budget of the system, and $c_{i}$ represents the contribution factor of feature $x_{i}$, typically determined by the task objective or reconstruction quality. In an ideal scenario, these factors exhibit certain patterns. However, in practical applications, the complexity of task objectives and reconstruction quality makes it difficult to directly obtain the contribution factors. In this paper, the gradient values are used as a measure of the feature contribution factors. Next, for each feature category, based on the allocated bit count $b_{k}$, the bits are divided into symbol bits $b_{k}^{0}$, integer bits $b_{k}^{1}$, and fractional bits $b_{k}^{2}$, with the constraint that $b_{k}^{0}=1$. The quantization step size formulas for the integer and fractional parts are as follows:

(17) $$\Delta_{k}^{1}=\frac{|x_{k}{|}_{\max}-{\left|x_{k}\right|}_{\min}}{2^{b_{k}^{1}-1}},\;\Delta_{k}^{2}=\frac{|x_{k}{|}_{\max}-{\left|x_{k}\right|}_{\min}}{2^{b_{k}^{2}-1}}$$

For each feature $x_{k}^{i}$ in the category, the corresponding quantization step size is used to quantize the feature, and the integer and fractional parts are combined to yield the final quantized feature value ${\tilde{x}}_{k}^{i}$. The expression is as follows:

(18) $${\tilde{x}}_{k}^{i}=\Delta_{k}^{1}\times \left\lfloor \frac{|x_{k}^{i}\left|-\right|x_{k}{|}_{\min}}{\Delta_{k}^{\prime }}+\frac{1}{2}\right\rfloor \cdot L\left(b_{k}^{\prime \prime }\right)+\Delta_{k}^{2}\times \left\lfloor \frac{|x_{k}^{i}\left|-\right|x_{k}{|}_{\min}}{\Delta_{k}^{\prime \prime }}+\frac{1}{2}\right\rfloor$$

where L(·) represents the left shift operation, which is used to combine the integer and fractional parts according to their allocated bit precision. Algorithm 1 summarizes the entire workflow of the non-uniform quantization algorithm.

**Algorithm 1** Contribution factor-based non-uniform quantization.
1:**Input:** Feature set *X*, contribution factors *C*, total bit budget *B*, number of categories *m*.2:**Output:** Quantized feature set $\tilde{X}$.3:
**Step 1: Feature classification**
4:**for** each feature $x_{i}$ in *X* **do**5:    Assign contribution factor $c_{i}$ based on task or reconstruction quality.6:    Classify features into *m* categories based on $c_{i}$.7:
**end for**
8:
**Step 2: Bit allocation**
9:**for** each category $D_{j}$ **do**10:    Allocate bits $b_{k}$ to each category based on the contribution factors:$$b_{k}=\left\lfloor \frac{\sum_{i=1}^{k}c_{i}}{\sum_{k=1}^{M}\sum_{i=1}^{k}c_{i}}\cdot B\right\rfloor .$$11:
**end for**
12:
**Step 3: Bit splitting**
13:**for** each category $D_{j}$ **do**14:    Split $b_{k}$ into symbol bits $b_{k}^{0}=1$, integer bits $b_{k}^{1}$, and fractional bits $b_{k}^{2}$,15:    Calculate the quantization step sizes $\Delta_{k}^{1}$ and $\Delta_{k}^{2}$:$$\Delta_{k}^{1}=\frac{|x_{k}{|}_{\max}-{\left|x_{k}\right|}_{\min}}{2^{b_{k}^{1}-1}},\;\Delta_{k}^{2}=\frac{|x_{k}{|}_{\max}-{\left|x_{k}\right|}_{\min}}{2^{b_{k}^{2}-1}}.\;$$16:
**end for**
17:
**Step 4: Quantization**
18:**for** each feature $x_{k}^{i}$ in $D_{j}$ **do**19:    Quantize the feature using $\Delta_{k}^{1}$ and $\Delta_{k}^{2}$:$$\tilde{x_{k}^{i}}=\Delta_{k}^{1}\cdot \left\lfloor \frac{|x_{k}^{i}\left|-\right|x_{k}{|}_{\min}}{\Delta_{k}^{1}}+0.5\right\rfloor \cdot L\left(b_{k}^{2}\right)+\Delta_{k}^{2}\cdot \left\lfloor \frac{|x_{k}^{i}\left|-\right|x_{k}{|}_{\min}}{\Delta_{k}^{2}}+0.5\right\rfloor .$$20:
**end for**
21:
**Step 5: Output**
22:Return quantized feature set $\tilde{X}$.


At the receiver end, the received quantized values need to be dequantized. First, the received quantized feature values are separated into integer and fractional parts according to the given integer bits $b_{k}^{1}$ and fractional bits $b_{k}^{2}$. Then, based on the quantization step sizes for the integer and fractional parts, they are dequantized. The restored integer and fractional parts are combined to form the final dequantized feature value ${\hat{x}}_{k}^{i}$. The detailed dequantization algorithm is shown in Algorithm 2.
**Algorithm 2** Dequantization algorithm for contribution factor-based non-uniform quantization.1:**Input:** Quantized feature set $\tilde{X}$, feature categories $D_{1},D_{2},\ldots ,D_{m}$, contribution factors *C*, total bit budget *B*, quantization step sizes $\Delta_{k}^{1}$, $\Delta_{k}^{2}$.2:**Output:** Restored feature set *X*.3:**Step 1: Bit splitting and inverse bit allocation**4:**for** each feature category $D_{j}$ **do**5:    Split each category’s total bit allocation $b_{k}$ into symbol bits $b_{k}^{0}$, integer bits $b_{k}^{1}$, and fractional bits $b_{k}^{2}$,6:    **Input:** Inverse quantization step sizes $\Delta_{k}^{1}$ and $\Delta_{k}^{2}$ from the sender.7:**end for**8:**Step 2: Dequantization of each feature**9:**for** each quantized feature $\tilde{x_{k}^{i}}$ in $D_{j}$ **do**10:    Extract the integer part and fractional part from $\tilde{x_{k}^{i}}$ using the left shift operation $L(b_{k}^{2})$:$${\hat{x}}_{\mathrm{int}}=\left\lfloor \frac{\tilde{x_{k}^{i}}}{L(b_{k}^{2})}\right\rfloor ,$$$${\hat{x}}_{frac}={\tilde{x}}_{k}^{i}\;\mathrm{mod}\;L\left(b_{k}^{2}\right).$$11:    Restore the original value for integer and fractional parts:$$x_{\mathrm{int}}={\left|x_{k}\right|}_{\min}+{\hat{x}}_{\mathrm{int}}\cdot \Delta_{k}^{1},$$$$x_{\mathrm{frac}}={\left|x_{k}\right|}_{\min}+{\hat{x}}_{frac}\cdot \Delta_{k}^{2}.$$12:    Combine the integer and fractional parts:$$x_{k}^{i}=x_{\mathrm{int}}+x_{\mathrm{frac}}.$$13:**end for**14:**Step 3: Output**15:Return the restored feature set $X=\{x_{1},x_{2},\ldots ,x_{n}\}$.

To preserve the gradient information during the quantization process, this paper adopts the straight-through estimator (STE). Using the STE technique, during backpropagation, an approximate gradient is used to replace the non-differentiable part of the quantization process, ensuring that the model can be properly optimized.

## 4. Experiments and Discussion of Results

In this section, we first introduce the experimental setup, including the dataset, comparison methods, and experimental details. Finally, the experimental results are presented and discussed.

### 4.1. Experiment Settings

Experimental environment: The experiments were conducted on a Windows 11 64-bit operating system. The computer was equipped with an Intel i9-12700H processor, an NVIDIA GeForce RTX4060 graphics card, and 8 GB of RAM. The project environment was based on the PyTorch framework and implemented in Python 3.9.

Datasets: The experiments were conducted using the CIFAR10 dataset, consisting of 10 categories of color images, with each category containing 6,000 images, for a total of 60,000 images. In addition, we evaluated our approach on the CIFAR100 dataset and Tiny ImageNet. CIFAR100 comprises 100 categories, with 600 images per category, totaling 60,000 images, offering greater diversity in image classes compared to CIFAR10. Tiny ImageNet consists of 200 categories, with 500 images per category, totaling 100,000 images, providing increased variation in both content and complexity while remaining computationally feasible within the scope of our experimental setup. During training, all images from the datasets were randomly cropped into 256 × 256 patches to ensure consistency across evaluations.

Evaluation metrics: To evaluate image reconstruction quality, we analyzed it from two perspectives, namely, image fidelity and perceptual quality, using the following three key metrics: PSNR, MS-SSIM, and LPIPS.

On the one hand, PSNR and MS-SSIM served as metrics for assessing image fidelity, quantifying the consistency between the reconstructed image and the original image by measuring pixel-wise differences and structural similarity. For PSNR, we used mean squared error (MSE) as the loss function to optimize the model. MSE is defined as the squared difference between the pixel values of the original and reconstructed images, and PSNR is derived by applying a logarithmic transformation to map MSE to a signal-to-noise ratio, expressed in decibels (dB). For MS-SSIM, we used the multi-scale structural similarity (MS-SSIM) index as the loss function to guide model training. In the result analysis, we report the absolute value of MS-SSIM to ensure consistent comparison and analysis with other metrics such as PSNR.

On the other hand, to evaluate the perceptual quality of the generated images, we introduced learned perceptual image patch similarity (LPIPS), a deep learning-based metric. Specifically, for both the original and generated images, we used a pre-trained VGG model to extract multi-level deep features. The perceptual difference between the images was then quantified by calculating the weighted $L_{2}$ distance in the feature space. In this study, we used LPIPS only as an evaluation metric to assess the perceptual similarity in image reconstruction quality.

Training U: We used an end-to-end approach to train the model as a whole. The training process was divided into two stages. In the first stage, we trained the model to effectively extract feature representations of the image. Here, we used its variant, Swin Transformer, as the semantic encoding module for the image. The parameter settings are the same as those proposed in [22], namely $\left[N_{1};N_{2}\right]=\left[2;4\right]$, $\left[C_{1};C_{2}\right]=\left[128;256\right]$, with a window size of 2. In the second stage, we loaded the parameters trained in the first stage and applied the method designed in this paper to process the semantic encoded image features. To reduce computational complexity, we set the feature category m=3. We explored the effects of different bit allocation strategies on the reconstructed image quality when the total bit count *B* was fixed. We use the Adam optimizer with a learning rate of $1\times 10^{-4}$ and a batch size of 128.

### 4.2. Results and Analysis

**Figure 3:**
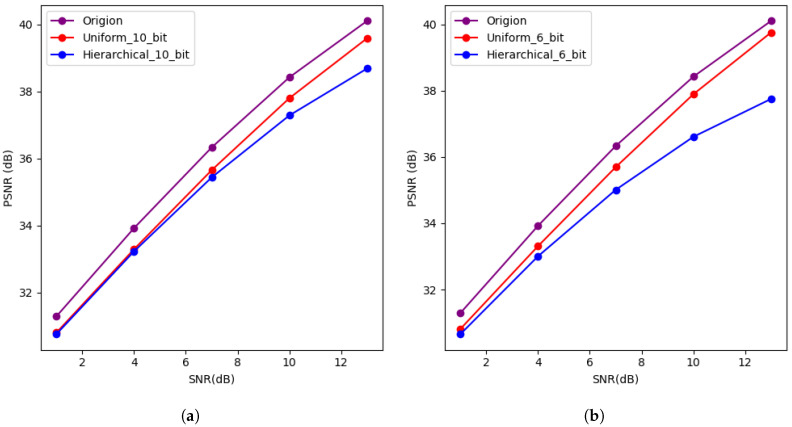

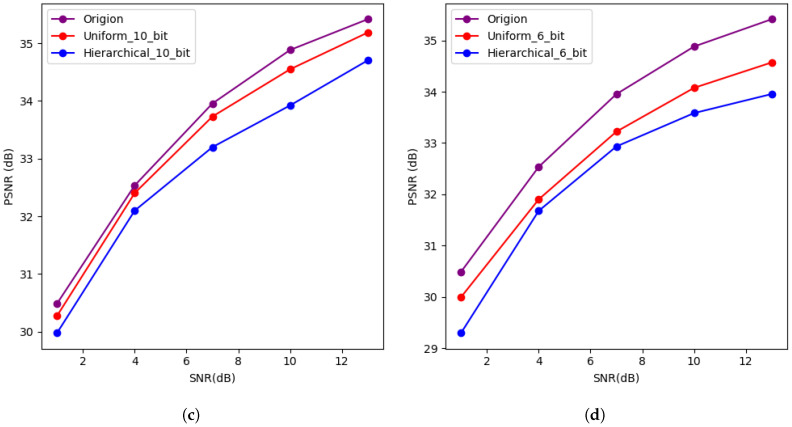
(**a**,**b**) Describes the image reconstruction PSNR values and MS-SSIM for the CIFAR10 dataset under the AWGN channel, with features transmitted using 10-bit uniform quantization, 6-bit uniform quantization, and no feature compression. (**c**,**d**) Show the PSNR values for image reconstruction of the CIFAR10 dataset under the Rayleigh channel.

Through comparative experiments, the performance of the proposed model under different channel conditions and compression ratios was validated. Figure 3 depicts a comparison of image recovery quality on the AWGN channel without considering bandwidth limitations during transmission. “Origin” represents the PSNR performance when no quantization is applied to the features. “Uniform_10” indicates the PSNR value when 10-bit quantization is applied to all features. “Hierarchical_10” represents the PSNR value when all features are divided into three feature levels, and 10-bit quantization is applied. The bit allocation for the three feature levels, based on their contribution factors, is set as follows: 5, 3, and 2. The division of the symbol bits, integer bits, and fractional bits is as follows: 5:131, 3:111, 2:110.

**Figure 4:**
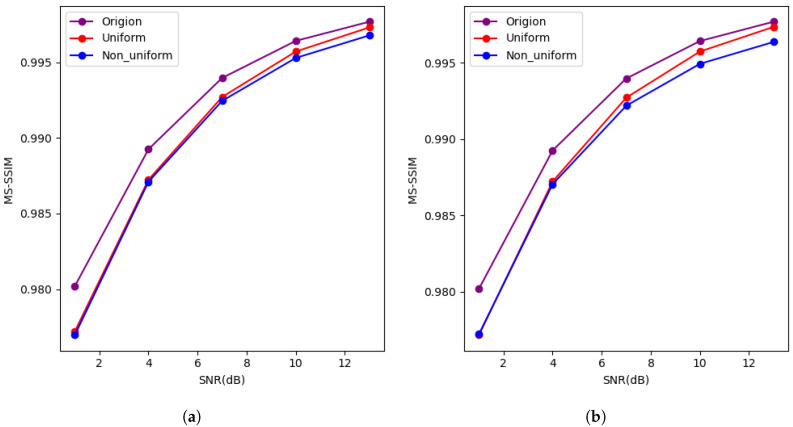

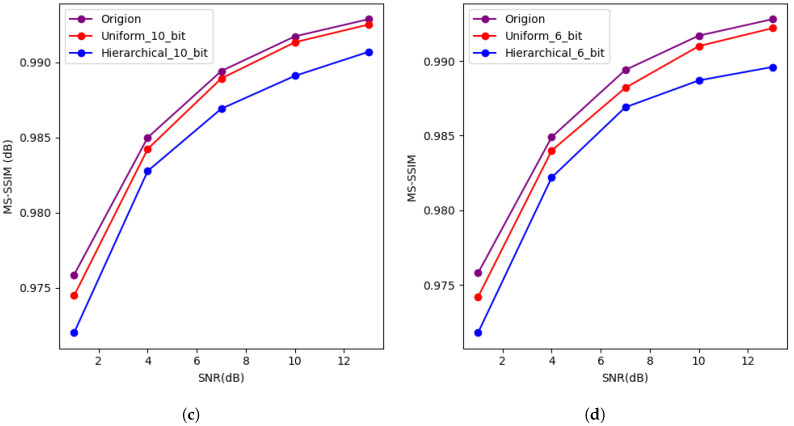
(**a**,**b**) Describe the image reconstruction MS-SSIM values for the CIFAR10 dataset under the AWGN channel, with features transmitted using 10-bit uniform quantization, 6-bit uniform quantization, and no feature compression. (**c**,**d**) Describe the MS-SSIM values for image reconstruction of the CIFAR10 dataset under the Rayleigh channel.

As shown in Figure 4, after quantizing the features, the image recovery quality at the receiver is noticeably affected compared to the case without quantization. As the available bandwidth increases (i.e., as the bit count per feature increases), the impact of quantization gradually diminishes. In both the AWGN and Rayleigh channels, at low signal-to-noise ratios (SNRs), the image recovery quality for both uniform and non-uniform quantization methods is similar. However, in the Rayleigh channel, the non-uniform quantization method slightly underperforms. At high SNR values, the image recovery quality still lags behind that of the unquantized case, but this gap diminishes, possibly due to the image recovery quality falling within an acceptable range.

To comprehensively evaluate the performance of the proposed model, we also tested and trained our model using the MS-SSIM metric. MS-SSIM is a commonly used image quality assessment metric that performs a weighted calculation of brightness, contrast, and structural similarity across multiple scales, providing a more accurate reflection of the perceptual quality of the image. The MS-SSIM performance of the model under different conditions is illustrated in Figure 4.

As presented in Figure 4, with the increase in bandwidth, the MS-SSIM values of different quantization methods gradually increase. The model proposed in this paper performs better in the AWGN channel compared to the Rayleigh channel.

To evaluate the perceptual quality of the image reconstruction, we used the LPIPS metric, which assesses the perceptual similarity between the reconstructed and original images. The results are shown in the figures below.

**Figure 5:**
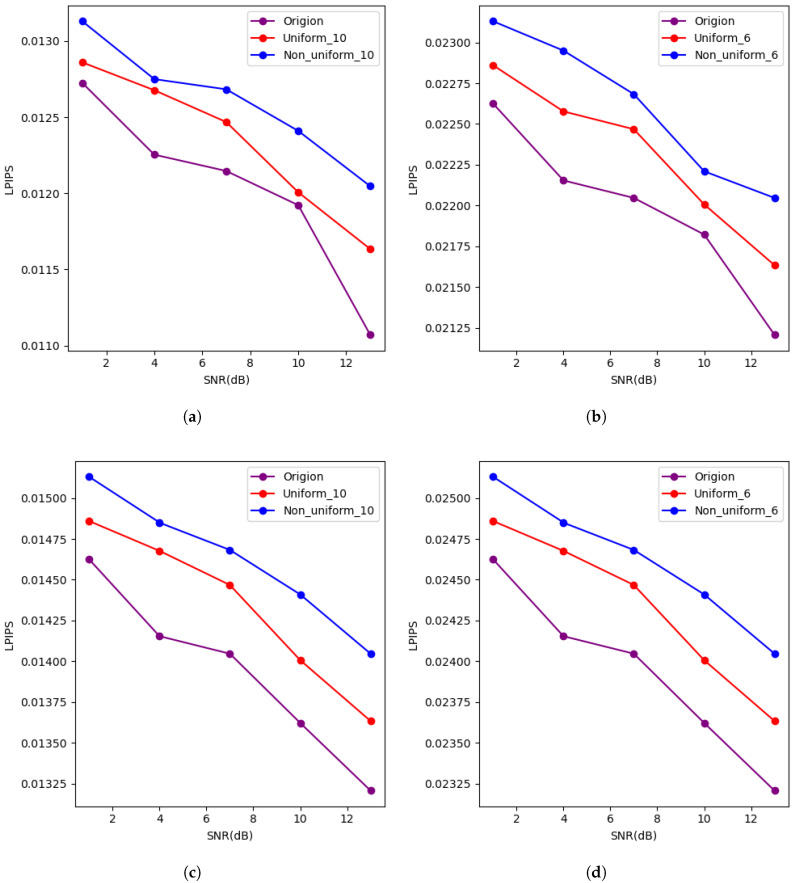
(**a**) Shows the LPIPS values for image reconstruction of the CIFAR10 dataset under the AWGN channel with no bandwidth limitations, comparing three methods: 10-bit uniform quantization for each feature, 10-bit non-uniform quantization, and no feature quantization. (**b**) Presents the LPIPS values for the CIFAR10 dataset under the AWGN channel with no bandwidth limitations, comparing the same three methods: 6-bit uniform quantization, 6-bit non-uniform quantization, and no feature quantization. (**c**) Describes the LPIPS values for image reconstruction of the CIFAR10 dataset under the Rayleigh channel with no bandwidth limitations, comparing 10-bit uniform quantization for each feature, 10-bit non-uniform quantization, and no feature quantization. (**d**) Shows the LPIPS values for the CIFAR10 dataset under the Rayleigh channel with no bandwidth limitations, comparing 6-bit uniform quantization, 6-bit non-uniform quantization, and no feature quantization.

Figure 5 shows the LPIPS values for image reconstruction of the CIFAR10 dataset under both AWGN and Rayleigh channels using different quantization methods. As the SNR increases, the LPIPS values decrease for all methods, indicating better perceptual quality of the reconstructed images. Both uniform and non-uniform quantization methods have higher LPIPS values compared to the unquantized method, as quantization inevitably affects image quality. However, the LPIPS values for all three methods are below the threshold for high-quality image recovery (0.05). This indicates that the proposed methods in this paper maintain the perceptual quality of the reconstructed images within an acceptable range, despite a slight decrease in quality.

**Figure 6:**
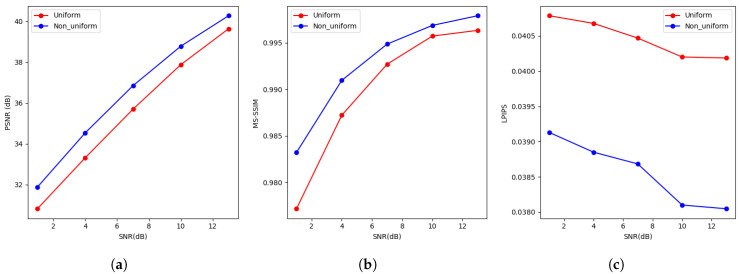
(**a**) Describes the relationship between PSNR and SNR under different quantization methods (Uniform and Non_uniform) in an AWGN channel with an 8-bit transmission limit. (**b**) Describes the relationship between MS-SSIM and SNR under different quantization methods (Uniform and Non_uniform) in an AWGN channel with an 8-bit transmission limit. (**c**) Shows the effect of 8-bit transmission on CIFAR10 images in an AWGN channel in terms of LPIPS.

Figure 6 presents the reconstruction results for the CIFAR10 dataset over an AWGN channel with 8-bit transmission. The experimental findings reveal that, under the constraint of limited transmission bits, the proposed non-uniform quantization method outperforms the uniform quantization method across the PSNR, MS-SSIM, and LPIPS metrics.

Specifically, in the PSNR metric (Figure 6a), non-uniform quantization shows significant improvement, particularly at higher SNR values. In terms of the MS-SSIM metric (Figure 6b), the non-uniform quantization method consistently maintains higher values compared to the uniform quantization method, indicating its superior ability to preserve structural details. Additionally, the LPIPS metric (Figure 6c) demonstrates that non-uniform quantization results in fewer perceptual differences from the original image, emphasizing its effectiveness in preserving perceptual quality.

To assess the robustness of our method in real wireless environments, we designed a composite channel model with multipath fading and time-varying noise. This model combines Rician fading (K=3), which includes a dominant line-of-sight component, and time-varying noise modulated as a sinusoidal waveform (sin(t/10), where t∈[0,100]) to simulate dynamic signal-to-noise ratio (SNR) changes. This model reflects more realistic conditions in daily wireless communication compared to simplified AWGN and Rayleigh models. We conducted a small-scale experiment using this real channel model to evaluate the performance of our method. In the experiment, the CIFAR-100 and Tiny ImageNet datasets were tested at SNRs of 4 dB and 10 dB, with SBR values of 1, 4, 7, 10, and 13, with a bit budget of 8 bits.

**Figure 7:**
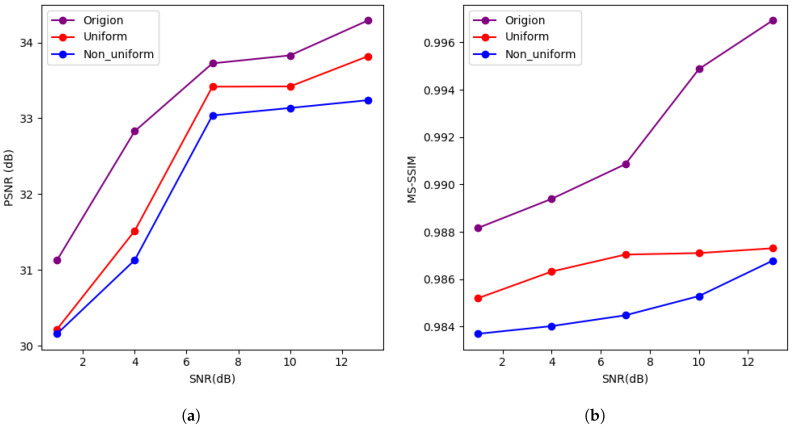

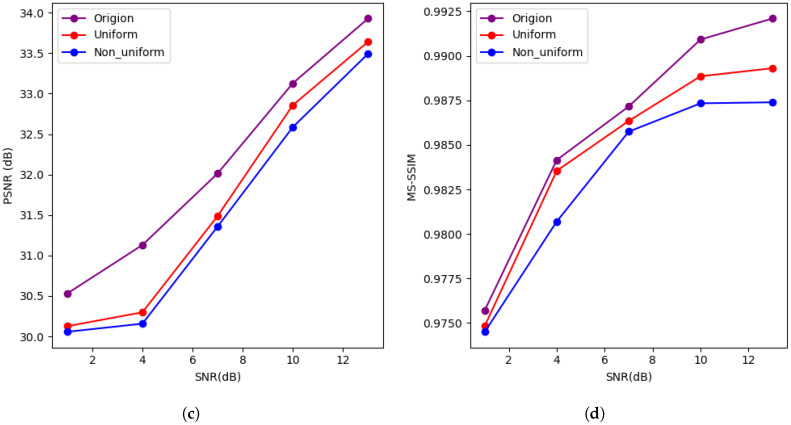
(**a**) Describes the relationship between PSNR and SNR for the CIFAR100 dataset under the composite channel with 8-bit transmission. (**b**) Describes the relationship between MS-SSIM and SNR for the CIFAR100 dataset under the composite channel with 8-bit transmission. (**c**) Describes the relationship between PSNR and SNR for the Tiny ImageNet dataset under the composite channel with 8-bit transmission. (**d**) Describes the relationship between MS-SSIM and SNR for the Tiny ImageNet dataset under the composite channel with 8-bit transmission.

**Figure 8:**
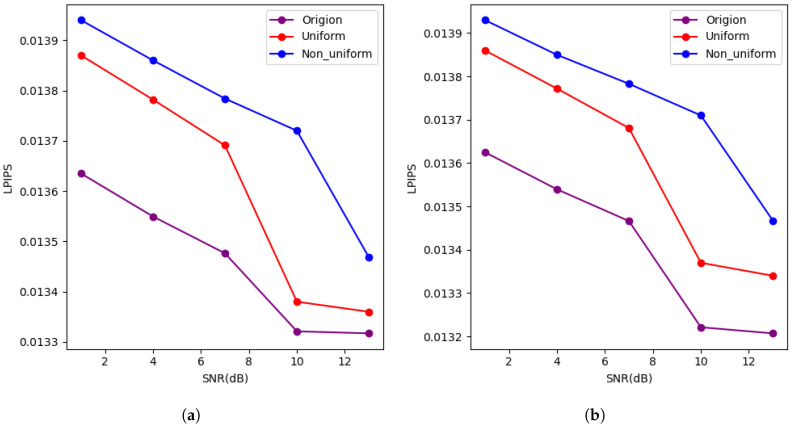
(**a**) Describes the relationship between LPIPS for the CIFAR100 dataset under the composite channel with 8-bit transmission. (**b**) Describes the relationship between LPIPS for the Tiny ImageNet dataset under the composite channel with 8-bit transmission.

The results (shown in Figure 7 and Figure 8) show that at low SNR, such as SNR = 1 dB, our method achieves a minimum PSNR of 30 dB, an MS-SSIM of 0.97, and an LPIPS value below 0.2, which indicates good image reconstruction quality. At high SNR, while our method achieves an MS-SSIM of 0.98 and an LPIPS value below 0.014, the PSNR reaches only 33 dB. This PSNR value, although acceptable, is still relatively low, which may result in some loss of fine details and image quality. Therefore, there is still room for improvement in terms of preserving detailed structures and achieving higher-quality image restoration.

Table 1 shows the image reconstruction quality of the Tiny ImageNet dataset under AWGN, Rayleigh, and composite channels with 8-bit transmission.

The results indicate that while there is a decrease in performance in the composite channel compared to the AWGN and Rayleigh channels, the image quality remains within an acceptable range. Specifically, the PSNR value in the composite channel (30.10 dB) is lower than in both the AWGN (33.12 dB) and Rayleigh (32.07 dB) channels, reflecting a slight reduction in reconstruction quality. However, the LPIPS value for the composite channel (0.0133) is very close to that of the AWGN channel (0.0135), suggesting that the perceptual quality of the reconstructed image is still maintained at an acceptable level.

### 4.3. Computational Overhead Analysis

To assess the computational feasibility of our non-uniform quantization scheme, which incorporates feature classification and bit-allocation logic, we conducted a profiling analysis and compared it to uniform quantization. This evaluation was conducted on the Tiny ImageNet dataset using test images under an AWGN channel (SNR = 10 dB, 8-bit transmission), consistent with the experimental setup used in previous sections. We measured the training time per epoch (epoch = 128), inference time per image, and peak memory usage for both methods.

For training, we profiled the full pipeline, including feature extraction, gradient-based contribution factor computation, bit allocation, and quantization. For inference, we measured the end-to-end process from the input image to the reconstructed output. The results are presented in Table 2. Compared to uniform quantization, our non-uniform approach increases training time by 20% (from 30 minutes to 36 minutes per epoch). Inference time rose by 12% (from 0.175 seconds to 0.195 seconds per image). Peak memory usage increased by 7.6% (from 6.5 GB to 7.0 GB). Despite these increases, our approach remains practical for resource-limited systems, especially when considering the gains in bandwidth efficiency under fixed bit budgets.

### 4.4. Ablation Studies on Feature Weighting

To evaluate the effectiveness of our gradient-based weighting approach for determining contribution factors, we conducted an ablation study, comparing it with two simpler heuristics, namely, the largest magnitude and the standard deviation of feature values. The experiment was performed on the Tiny ImageNet datasets, using both an AWGN ideal channel (SNR = 10 dB, 8-bit transmission) and the designed composite channel. Reconstruction quality was evaluated using PSNR and LPIPS. As shown in Table 3, our gradient-based method achieved a PSNR of 34.52 dB and an LPIPS of 0.028, outperforming the largest magnitude heuristic (PSNR = 30.25dB, LPIPS = 0.046) and the standard deviation heuristic (PSNR = 32.12 dB, LPIPS = 0.054). Similarly, under the composite channel, our method also outperformed the heuristics in both PSNR and LPIPS, as shown in Table 4. These results demonstrate that our gradient-based approach effectively captures contextual dependencies, leading to better accuracy and perceptual quality compared to the simpler heuristics in both channels.

## 5. Conclusions

In this paper, we propose a semantic communication system for image transmission, which utilizes a non-uniform quantization method and feature contribution factors to achieve efficient transmission under bandwidth constraints. The core of the system is a non-uniform quantization algorithm that dynamically allocates bit rates based on the contribution of each feature. This approach enhances both image transmission quality and compression performance. Additionally, we present an implementation of this non-uniform quantization system for image transmission, based on the Swin Transformer. To validate the feature importance measurement based on contribution factors, we conducted ablation experiments comparing our method with other approaches. Under limited bandwidth and varying signal-to-noise ratio (SNR) conditions, the experimental results demonstrate that our method outperforms both unquantized and uniform quantization approaches. The proposed quantization framework effectively balances image reconstruction quality and compression efficiency, providing reliable support for semantic communication systems.

## 6. Future Work

While our current framework focuses on single-user image transmission, the non-uniform quantization approach based on feature contribution factors offers promising extensions to broader scenarios. Below, we outline several directions for future research, which are based on potential extensions of our approach to multi-user systems, multimodal data, and dynamic network environments.

In multi-user scenarios, our method could be extended through a joint optimization strategy. By considering individual user requirements (such as image quality preferences) and network conditions (such as available bandwidth), we can dynamically allocate bits and bandwidth across users. This would ensure fair resource distribution while maintaining transmission efficiency, and could potentially incorporate game-theoretic or fairness-based optimization techniques.

For multimodal data, such as combined image and audio inputs, our approach can evolve by incorporating multimodal feature selection. By jointly assessing the contribution factors of features across modalities, we can optimize quantization bits to prioritize critical information (such as key visual details or audio cues). This would enable efficient cross-modal transmission, particularly suited for tasks like multimedia streaming or multimodal perception.

In dynamic network environments, real-time adaptive bit allocation could be optimized through the integration of reinforcement learning. This would enable the system to adjust quantization thresholds dynamically based on fluctuating channel conditions (e.g., signal-to-noise ratio under AWGN or Rayleigh fading), ensuring a balance between image quality and bandwidth usage. Such an approach would enhance robustness and adaptability in practical deployments.

Another important extension in future work will focus on dynamic bandwidth variations. We plan to explore how fluctuations in available bandwidth can affect the performance of our system. By incorporating real-time bandwidth adaptation, we can further optimize the system’s ability to maintain image quality and transmission efficiency under conditions of varying available bandwidth, simulating more realistic scenarios encountered in mobile or dynamic environments.

These extensions will broaden the applicability of our feature-driven semantic communication framework, addressing diverse real-world challenges. Future work will further explore these directions through both theoretical modeling and experimental validation. In addition, beyond current datasets (CIFAR-10, CIFAR-100, Tiny ImageNet), we also plan to validate our method on larger, more complex datasets like ImageNet and COCO, which include high-resolution images and diverse scenes, to further test its scalability in real-world semantic communication scenarios.

## Figures and Tables

**Table 1:** Performance across channels (SNR = 4 dB).

Channel	PSNR (dB)	LPIPS	MS-SSIM (dB)
AWGN	33.12	0.0135	0.982
Rayleigh	32.07	0.021	0.984
Composite	30.10	0.0133	0.981

**Table 2:** Computational overhead comparison (Tiny ImageNet, SNR = 10 dB).

Method	Training Time (min/epoch)	Inference Time (s/image)	Peak Memory (GB)
Uniform Quantization	30	0.175	6.5
Non-Uniform Quantization (Ours)	36	0.195	7.0

**Table 3:** Ablation study results (SNR = 10 dB, Tiny ImageNet, AWGN channel).

Weighting Method	PSNR (dB)	LPIPS	MS-SSIM	Inference Time (s)
Gradient-based (Ours)	34.52	0.028	0.975	0.195
Largest magnitude	30.25	0.046	0.925	0.143
Standard deviation	32.12	0.054	0.930	0.162

**Table 4:** Ablation study results (SNR = 10 dB, Tiny ImageNet, composite channel).

Weighting Method	PSNR (dB)	LPIPS	MS-SSIM	Inference Time (s)
Gradient-based (Ours)	32.45	0.035	0.960	0.215
Largest magnitude	28.55	0.062	0.910	0.175
Standard deviation	30.47	0.070	0.920	0.190

## Data Availability

Data are contained within the article.
